# A Bidirectional Relationship between Executive Function and Health Behavior: Evidence, Implications, and Future Directions

**DOI:** 10.3389/fnins.2016.00386

**Published:** 2016-08-23

**Authors:** Julia L. Allan, David McMinn, Michael Daly

**Affiliations:** ^1^Health Psychology, Institute of Applied Health Sciences, University of AberdeenAberdeen, UK; ^2^Behavioural Science Centre, Stirling Management School, University of StirlingStirling, UK; ^3^UCD Geary Institute, University College DublinDublin, Ireland

**Keywords:** executive function, cognitive ability, health behavior, physical activity, substance use, diet, health

## Abstract

Physically active lifestyles and other health-enhancing behaviors play an important role in preserving executive function into old age. Conversely, emerging research suggests that executive functions facilitate participation in a broad range of healthy behaviors including physical activity and reduced fatty food, tobacco, and alcohol consumption. They do this by supporting the volition, planning, performance monitoring, and inhibition necessary to enact intentions and override urges to engage in health damaging behavior. Here, we focus firstly on evidence suggesting that health-enhancing behaviors can induce improvements in executive function. We then switch our focus to findings linking executive function to the consistent performance of health-promoting behaviors and the avoidance of health risk behaviors. We suggest that executive function, health behavior, and disease processes are interdependent. In particular, we argue that a positive feedback loop may exist whereby health behavior-induced changes in executive function foster subsequent health-enhancing behaviors, which in turn help sustain efficient executive functions and good health. We conclude by outlining the implications of this reciprocal relationship for intervention strategies, the design of research studies, and the study of healthy aging.

## Introduction

Physical activity has been described as “the best buy in medicine” (Loprinzi, 2015). Having an active lifestyle not only reduces the risk of chronic conditions (Warburton et al., 2006), it also helps preserve cognitive function (Sofi et al., 2011) and in particular, higher level **“executive” function** (EF) into old age (Colcombe and Kramer, 2003), and by doing so may enhance subsequent activity levels and foster a range of other health protective behaviors, producing long-run health benefits (Loprinzi, 2015). In the same way, engagement in other health behaviors could yield similarly reciprocal cognitive and behavioral benefits and reduce the incidence of chronic disease.

KEY CONCEPT 1 **What is Executive Function?**

EF is an umbrella term for the related but distinct processes involved in the effortful control of goal directed behavior. Largely mediated by the prefrontal cortex, EF has been summarized as “.… the co-ordinated operation of various processes to accomplish a particular goal in a flexible manner” (Funahashi, 2001) and encompasses three main factors:

- mental flexibility/set shifting (strategically shifting attention between goals and flexibly adapting thinking to suit the situation),
- monitoring and updating working memory (evaluating and updating goal relevant information held in mind), and
- inhibition of prepotent responses (suppressing inappropriate, dominant, or habitual responses when necessary).

In this review we propose that a set of self-reinforcing positive feedback loops link health-protective behaviors and EF. First, we provide an overview of research linking physical activity and other health-enhancing behaviors to increases in EF. Second, we examine emerging evidence pointing to EF as a key driver of the successful adoption and maintenance of physically active lifestyles and health promoting behavior. Third, we propose an integrated reciprocal account whereby EF and health behavior are linked through a feedback loop that could confer substantial health benefits over time. Finally, we outline implications for intervention strategies and study design.

In the context of cognitive function, higher-level EFs are of critical importance as they provide the self-regulatory resources needed to effectively plan and execute goal-directed behaviors (Hofmann et al., 2012b). Specifically, the EFs are the higher-order cognitive processes involved in the planning, initiation, sequencing and monitoring of complex goal directed behavior (Royall et al., 2002; Lezak et al., 2004) and can be thought of collectively as the processes which enable top-down control of behavior, thoughts, and emotion. While the concept is notoriously difficult to define, there is broad agreement that the term covers three distinct but overlapping processes: mental flexibility, working memory, and inhibition (Miyake et al., 2000; Miyake and Friedman, 2012).

Over the lifespan, the combined influence of these three facets of EF allows health promoting goals to be pursued despite the presence of distraction, tempting situational cues, and visceral desires (Hall and Marteau, 2014). EF typically declines in old age, potentially compounding age related declines in health as EF plays an important role in carrying out tasks including chronic illness management (Tomlin and Sinclair, 2016) and in allowing health-enhancing behaviors to be enacted and health-damaging behaviors to be resisted (Hall et al., 2008a; Williams and Thayer, 2009). Perhaps as a result, those with better EF tend to avoid chronic illness and live longer even following the diagnosis of a chronic illness (Duff et al., 2009; Hall et al., 2009, 2010). Fully understanding the cognitive factors associated with positive health behaviors and with healthy aging in general is vitally important in the context of our rapidly aging population. By 2050, the World Health Organization anticipates that 1.5 billion people will be aged 65 or over (World Health Organisation, 2011) so ensuring that health is preserved into old age is a key goal for health scientists.

In addition to influencing health outcomes, EFs are themselves influenced by health-related behaviors and the resulting disease processes in several ways. For example EF is less efficient in individuals who exercise less than others (Colcombe and Kramer, 2003), who are obese (Debette et al., 2011) or who show evidence of systemic inflammation (Komulainen et al., 2007; Trollor et al., 2012). We suggest that positive feedback loops exist whereby EF sustains health protective behaviors which in turn protect cognitive function and physical health into old age (McMinn et al., 2013). In our previously published paper (Daly et al., 2015), the catalyst for this review, we provided a demonstration of this idea in the area of physical activity, showing not only that higher levels of EF lead to increased future levels of physical activity, but also that higher levels of physical activity contribute to future improvements in EF.

This original paper was written in the context of (a) a substantial evidence base linking physical activity level to improvements in executive functioning, and (b) suggestions from the literature that the opposite may also be the case i.e., that efficient EF may facilitate future engagement in physical activity. Our study was designed to test this by modeling the directionality of the physical activity—EF relationship.

Using data collected from 4555 older adults over 6 years (4 study waves) of the English Longitudinal Study of Aging (ELSA), we ran three separate sets of analyses. Firstly, we examined the cross-sectional association between physical activity and executive functioning for individuals across the four study waves using multilevel modeling adjusting for age, sex, education, wealth, and long-standing illness. Next we examined how changes in physical activity related to simultaneous changes in executive functioning by conducting a fixed effects analysis to test whether within-person variation in physical activity was associated with within-person variation in executive functioning. By examining within-person variation in this way, any effects of non-observed time-invariant confounders (i.e., factors such as genetics, early adversity etc.) are essentially ruled out as they would not be expected to vary within people over time. Finally, we used longitudinal multilevel modeling to test whether physical activity at one point in time could be used to predict changes in EF and whether the efficiency of EF could predict subsequent engagement in physical activity.

These analyses revealed that physical activity and EF were closely interlinked and that the association between the two remained after controlling for demographic and health characteristics. Furthermore, we demonstrated that dynamic within-person changes in EF corresponded with parallel changes in physical activity, largely ruling out the possibility that non-observed, time-invariant confounders had produced the association. Critically, we found evidence that the relationship between physical activity and EF is bidirectional. Those with poor EF showed subsequent decreases in their rates of participation in physical activity and older adults who engaged in sports and other activities involving physical exertion tended to retain high levels of EF over time.

In the present review we describe in more detail the evidence supporting the existence of a bidirectional, reciprocal link between physical activity and EF and expand this idea to incorporate other health protective behaviors.

## Being physically active: Positive effects on cognitive function

With a rapidly aging population it becomes increasingly important to identify behaviors that people can engage in that may slow cognitive decline, or even improve cognitive abilities over time (Singh-Manoux et al., 2012). There is a large body of evidence demonstrating the beneficial effect of **physical activity** on EF. Colcombe and Kramer's highly influential (2003) meta-analysis of 18 randomized intervention studies found a larger beneficial effect on cognitive task performance for older adults who received exercise interventions [0.478 (*SE* = 0.029, *n* = 101, *p* < 0.01)] compared with those in control groups [0.164 (*SE* = 0.028, *n* = 96, *p* < 0.05)] (Colcombe and Kramer, 2003). When compared to other aspects of cognitive function, exercise had the greatest beneficial effect on higher level executive processes [*g* = 0.68 (*SE* = 0.052, *n* = 37, *p* < 0.05)]. It is important to note that the meta-analysis conducted by Colcombe and Kramer included only exercise interventions. Exercise is a sub-type of physical activity that has the distinct features of being planned and structured, with the goal of improving fitness (Hill et al., 2015). Exercise is typically associated with a higher intensity of physical activity than daily incidental activities, and may therefore confer greater cognitive benefits. In a subsequent meta-analysis including more recent studies but limiting inclusion to randomized controlled trials (*n* = 19), Smith et al. (2010) reported a beneficial but more modest effect of exercise on EF compared with the analysis of Colcombe and Kramer [*g* = 0.123 (95% CI:0.021 to 0.225), *p* = 0.018].

KEY CONCEPT 2 **Physical activity**

Physical activity is defined as “any bodily movement produced by skeletal muscle that results in energy expenditure” (Caspersen et al., 1985), and encompasses a broad range of activities from every day “incidental” activity such as active commuting or dog walking, to more structured, planned, purposive activities like team sports, or fitness classes.

More recently, reviews have sought to better understand this relationship in different age groups and for specific facets of EF. Guiney and Machado (2013) found that in older adults, intervention studies indicate that regular aerobic exercise can improve task switching, selective attention, inhibition of prepotent responses, and working memory span. Although they concluded that there is a paucity of studies in young adults and children, the limited available evidence supports a beneficial effect of physical activity on task switching, selective attention, inhibitory control, and working memory updating in young adults, and working memory capacity, selective attention and inhibitory control, in children. Kelly et al. (2014) conducted a meta-analysis investigating the effect of different modes of activity on a range of EF components. Resistance training was found to have beneficial effects on measures of reasoning, while Tai Chi improved measures of attention and processing speed.

Collectively these findings provide a convincing body of evidence to support the beneficial effects of physical activity on EF. A broad set of animal and human studies has also shed light on the neurobiological pathways through which increased activity levels improve cognitive function.

## From activity to better cognitive function: Potential mechanisms

**Figure 1:**
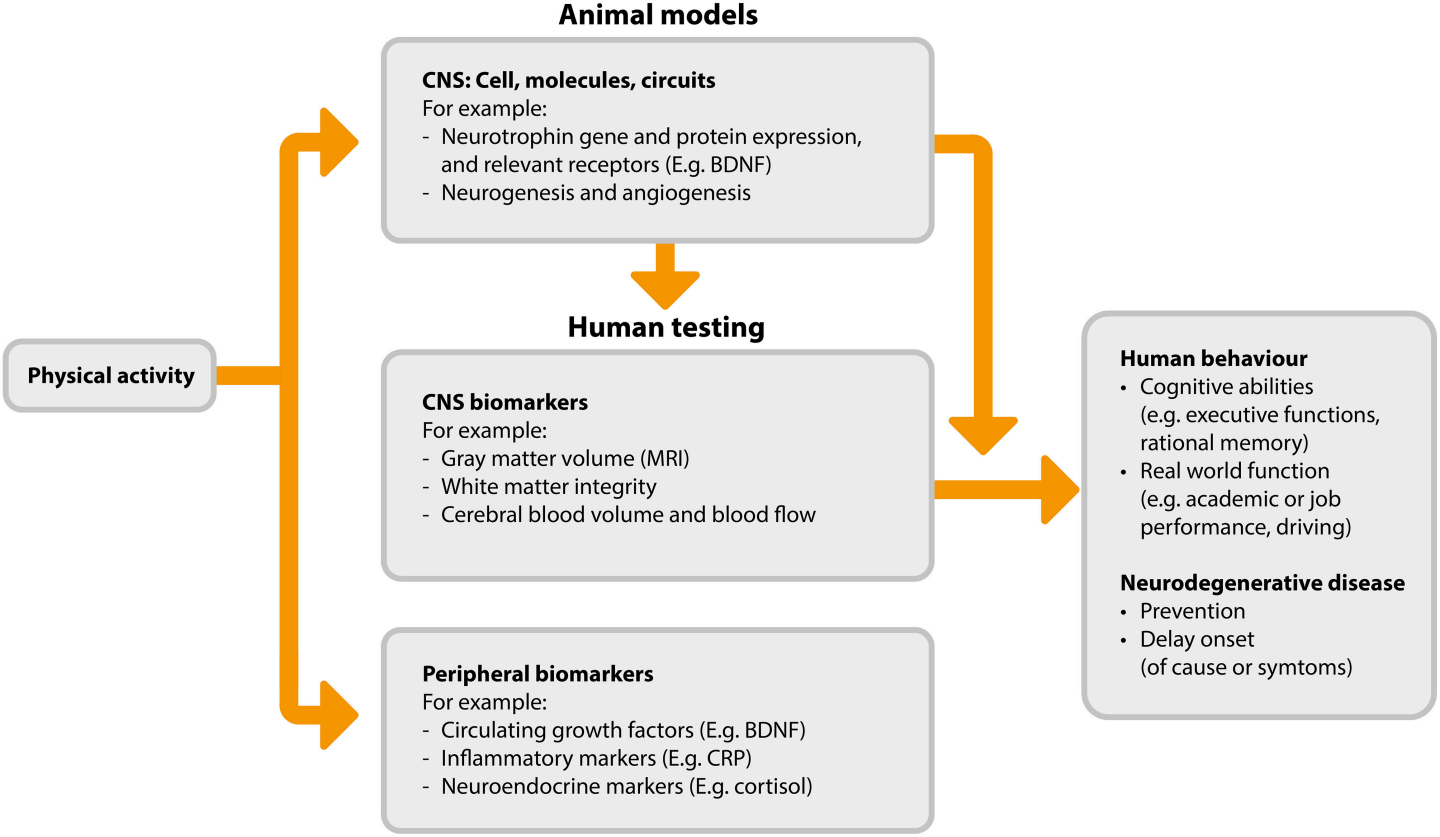
**Proposed pathways and measurements associated with physical activity induced cognitive benefits (adapted from Figure 2 in Voss et al., 2013b)**. BDNF, brain derived neurotrophic factor; CNS, central nervous system; CRP, C-reaction protein; MRI, magnetic resonance imaging.

A detailed review of the possible neurobiological processes driving the association between activity and cognitive function is beyond the scope of this review, and is available elsewhere (Voss et al., 2013b). However, we provide an overview of the mechanisms and mechanism measurements in Figure 1. The flow of this figure represents the direction of benefit from physical activity to improved cognitive abilities, while also highlighting that investigations begin with animal-based studies which serve to inform subsequent studies in humans. The proposed mechanisms by which physical activity may improve cognitive function fall broadly into three categories: (1) increased levels of neurotrophins, (2) neurogenesis and changes in brain structure, and (3) angiogenesis.

Neurotrophins are a class of growth factor consisting of proteins that bring about the survival, development, and function of neurons. Maintenance or increases in these neurotrophins are thought to confer neuroprotective benefits. One of the most abundant neurotrophins is brain-derived neurotrophic factor (BDNF). A number of animal studies have shown exercise-dependent changes in BDNF in the brain and upregulation of central BDNF expression in response to activity (Neeper et al., 1995, 1996; Liu et al., 2009; Hopkins et al., 2011; Marlatt et al., 2012; Hong et al., 2015). In humans, physical activity has been shown to increase circulating levels of BDNF—a proxy for central expression (Knaepen et al., 2010; Zoladz and Pilc, 2010; Kim et al., 2014; Leckie et al., 2014). Neurotrophins such as BDNF may promote cognitive benefits in several different ways, including by neural survival (Baydyuk and Xu, 2014) and improved synaptic plasticity (Leal et al., 2014), and also through the stimulation of neurogenesis (i.e., the formation of new neurons) and related changes in brain structure (Binder and Scharfman, 2004).

Neurogenesis is considered to be positive, and whilst neurogenesis continues throughout life it generally declines with age. In animals, physical activity brings about considerable growth of new neurons in both young and old brains (van Praag et al., 1999a,b; van Praag et al., 2005; Inoue et al., 2015; Sung, 2015; Nokia et al., 2016). In humans it is not possible to study neurogenesis at the cellular level using non-invasive techniques. However, imaging techniques allow for the investigation of the effect of physical activity on brain structure and integrity via changes in gray and white matter. Studies using such imaging techniques have observed physical activity-associated increases in hippocampal volume (Erickson et al., 2011; Niemann et al., 2014; Thomas et al., 2015), gray matter volume in the prefrontal, frontal, and lateral temporal cortices (Colcombe et al., 2006; Flöel et al., 2010; Ruscheweyh et al., 2011; Köbe et al., 2015), and in frontal and temporal white matter integrity (Voss et al., 2013a; Tian et al., 2015; Zhu et al., 2015b). While this evidence is suggestive of physical activity-induced neurogenesis, we recognize that the imaging techniques used for such *in vivo* human studies rely on correlations to indirectly quantify neurogenesis, and it is likely that much of the observed growth may be due to angiogenesis, synaptogenesis, changes in myelin, and other neural growth processes in addition to the birth of new neurons.

Finally, physical activity may influence cognitive function via angiogenesis in the brain, i.e., the formation of blood vessels from existing vasculature. Brain angiogenesis is thought to be stimulated by increased blood flow to the brain during exercise, thus facilitating increased growth and functioning of many of the brain structures and processes responsible for cognition. Brain endothelial cell development (Lopez-Lopez et al., 2004) and angiogenesis (Kleim et al., 2002; Swain et al., 2003; Gao et al., 2014) are promoted in response to physical activity. The physiological mechanisms responsible for increased angiogenesis include elevated phosphorylation of endothelial nitric oxide synthase, raised levels of bone marrow-derived CD34+ cells, and subsequent endothelial or mechanical mechanisms leading to neovascularization and correction of endothelial dysfunction (Huber-Abel et al., 2012; Schmidt et al., 2013).

In addition to these three proposed mechanisms of physical activity-induced improvements in EF, there is also a body of *in vitro* and *ex-vivo* evidence to support a beneficial effect of physical activity on reduced inflammatory markers (Ertek and Cicero, 2012). Given that increased levels of inflammation are associated with reduced EF performance (Trollor et al., 2012), the inflammation-reducing effect of physical activity may be an additional mechanism by which physical activity benefits the EFs.

Regardless of the exact set of pathways which leads exercise to have a beneficial effect on cognitive health, it is apparent that engaging in physical activity brings positive cognitive consequences, particularly to the EFs. Physical activity, however, is part of a broader set of health behaviors capable of producing marked changes in executive functioning.

## Cognitive effects of diet, smoking, and drug use

Several large well-conducted studies have demonstrated that the positive influence of health behavior on the EFs extends beyond physical activity. For example, Valls-Pedret et al. (2015) randomized 446 participants (mean age, 70 years) over a 6 year period to follow different diets. Those allocated to eat a Mediterranean diet (high in fruit, vegetables, and fish) with the additional supplement of olive oil had significantly better EF post intervention than controls, as measured by the trail making test (a measure of flexible switching of attention). The cognitive benefits of this type of diet were also observed in a longitudinal study of 527 older Australian adults. Adherence to a Mediterranean-style diet was associated with reduced decline in EF scores at 36 months, measured using the Delis-Kaplan Executive Function System, whereas Western diet adherence (high in red meat, refined grains, dairy, sugar) was associated with greater cognitive decline at 36 months (Gardener et al., 2015). Similarly, greater monounsaturated fat (i.e., the “good” fat found in large quantities in the Mediterranean diet) intake has been associated with reduced cognitive decline after 3 years in a cohort of 842 women (Naqvi et al., 2011). Such effects appear to be robust over long time periods: Zhu et al. (2015a) conducted a 25-year longitudinal study of 2435 men and woman, assessing their diet quality at baseline and EF at years 5 and 25. They found that a higher quality diet (measured as high consumption of healthy foods and low consumption of unhealthy foods) was associated with better EF scores at year 5 and year 25 (as measured by the Stroop test). The mechanism by which healthy diets, and in particular the Mediterranean diet, positively influence cognitive health likely stems from the high levels of antioxidants and anti-inflammatory agents present in the foods that comprise these diets. These agents are thought to mitigate oxidative processes in the brain, thereby protecting against neurodegeneration, whilst simultaneously promoting neurogenesis and bolstering neurotrophic factors (Huhn et al., 2015).

In contrast to the cognitive-enhancing effects of a healthy diet, smoking and substance use appear to have negative effects on cognition. Smoking is reliably related to a decline in general cognitive function (Ott et al., 2004; Almeida et al., 2011; Corley et al., 2012; Mons et al., 2013). When the impact of smoking on the EFs in particular is considered, the evidence is more limited but in the direction anticipated. For example, in 127 healthy older adults, Razani et al. (2004) reported that heavy smokers performed more poorly on tests of EF than moderate smokers and light/never smokers, suggesting a possible dose-response relationship between smoking and EF. Similarly, Sabia et al. (2012) performed a longitudinal analysis of 5099 men and 2137 women and found a greater rate of decline in executive functioning for current male smokers compared with male never smokers [mean difference in 10-year decline = −0.11 (95% CI: −0.17; −0.05)]. Interestingly, they also observed that recent ex-smokers had greater cognitive decline [−0.08 (−0.14; −0.02)] than long-term ex-smokers (who had a similar rate of cognitive decline as never smokers) suggesting that the detrimental effect of smoking may be reversible. The damaging effect of smoking on the EFs may be explained in part by the loss of gray matter volume seen in smokers in the prefrontal cortex and the anterior cingulate cortex, areas critically involved in higher level cognitive processing (Almeida et al., 2011; Fritz et al., 2014).

Drug and alcohol use have also been linked with changes in executive functioning. In addition to the acute detrimental effects of alcohol on EF (Montgomery et al., 2011; Marinkovic et al., 2012), chronic alcohol consumption causes increased levels of executive dysfunction (Joyce and Robbins, 1991; Moselhy et al., 2001; Brokate et al., 2003), which are maintained even once individuals are detoxified (Kopera et al., 2012). Cannabis use has also been linked to both short-term temporary impairment to attentional and information processing abilities, and longer-term permanent consequences in relation to impaired decision making and increased propensity to engage in risk taking behaviors (Crean et al., 2011). Similarly, cocaine use is associated with impaired inhibition (Kaufman et al., 2003; Hester and Garavan, 2004), and heroin users demonstrate increased impulsivity and poor planning abilities (Pau et al., 2000; Baldacchino et al., 2015), all deficits indicative of impaired EF. This effect may well be cumulative as polysubstance users (i.e., individuals regularly taking more than one type of drug) exhibit greater EF impairment across multiple domains than others (Verdejo et al., 2004; Fernández-Serrano et al., 2010).

It is important to note that the causal nature of the proposed associations between drug use and cognitive impairment in humans requires further research as most studies to date have not adjusted for potential confounding factors that could predispose people to both drug use and cognitive decline (e.g., family environment, personality traits, genetic factors) (Daly, 2013; Jackson et al., 2016). However, a range of studies have pointed to mechanistic processes that may produce such impairments. For instance, alcohol-related cognitive decline has been linked to neuronal damage and decreased neurogenesis (Nixon and Crews, 2002; Tateno and Saito, 2008) and cannabis-related cognitive impairment has been associated with reduced hippocampal volume (Yücel et al., 2008) or dysfunctional brain activation in the left cerebellum and the right lateral orbitofrontal cortex (Bolla et al., 2005). Cocaine use has been associated with reduced gray matter concentration (Franklin et al., 2002; Matochik et al., 2003) and decreased regional cerebral blood flow (Ernst et al., 2000) caused by constriction of coronary and cerebral vessels.

In this section we have shown how certain health behaviors (namely physical activity, diet, and drug use) can both positively and negatively impact executive functioning. Understanding the impact that these different health behaviors have on executive functioning is important in itself, however, improved EF is not necessarily the endpoint. In all likelihood, changes in EF strength or efficiency will lead to associated changes in the ability to engage in planned health-related behaviors in future, that is, EF may be both a consequence of and a predictor of further engagement in healthy behaviors. In the following section we look more closely at the role of the executive functions for initiating and maintaining health-related behaviors.

## Executive function as a determinant of healthy behavior

In addition to *benefiting from* engagement in healthy behaviors such as physical activity and healthy diet, EF is also likely to be *necessary for* the initiation and maintenance of such behaviors. Healthy behaviors typically involve short-term costs in the pursuit of longer-term benefits (Chapman, 2005). Important outcomes such as weight loss, fitness and health are all achieved by effortfully and consistently changing behavior in the present, e.g., by effortfully eating healthy foods, by not smoking, by drinking less alcohol, by going for a walk, and so on. Consequently, it is logical that good EF—the capacity to effortfully and strategically control behavior in pursuit of future goals—should improve the chances that people will be able to initiate and maintain health-relevant behaviors in order to obtain health outcomes.

Theoretical models of EF suggest that EF will be heavily involved in any situation requiring (1) planning and decision-making, (2) error monitoring and correction, (3) sequencing of actions, (4) complex actions, (5) inhibition of habitual responses or resistance to attractive stimuli, or (6) novel actions (Norman and Shallice, 1986). With this in mind, it is clear why EF is likely to be involved in the enactment of health protective behaviors. Taking the example of physical activity: activities must be planned, decisions must be made about when and where to be active, failures of activity intentions (e.g., failures to cycle to work, periods of sedentary behavior) must be detected and compensated for to maintain progress toward the goal, actions must be sequenced (e.g., alarm clock set earlier, appropriate clothing put on, change of clothes packed for work, etc), temptation from enjoyable but sedentary activities (e.g., watching tv) must be resisted, and solutions to novel problems (e.g., how to be active if weather precludes the planned activity) must be generated.

This intuitive role of EF in the initiation and maintenance of effortful health behaviors has been formalized in Hall and Fong's **Temporal Self-Regulation Theory** (TST; Hall and Fong, 2007, 2013, 2015). TST proposes that an individual's ability to engage in health behaviors that have short-term costs and longer-term benefits (like dieting or going to the gym) will be directly related to their natural executive control ability, and in particular to their ability to inhibit prepotent responses. Specifically, people with strong EF will be more likely to turn their healthy intentions into action, as they will be more able to resist temptation/suppress previous habits/inhibit responses to unhelpful cues in the environment. Such individuals may also be more likely to avoid temptation in the first place (Hofmann et al., 2012a), again improving the chances that intentions will be successfully enacted. When such ideas are empirically tested, individual differences in EF do indeed seem to moderate the strength of association between intentions and health behaviors (Hall et al., 2008b). Current dieters who have strong response inhibition are more likely to successfully resist temptation and to lose weight than those with similar diet goals but weak response inhibition (Hofmann et al., 2014). Smokers who show increased activation in brain regions consistently associated with response inhibition and conflict detection (right inferior frontal gyrus, pre supplementary motor area, and basal ganglia) during a response inhibition task are less likely to smoke in response to cravings than others (Berkman et al., 2011). Individuals scoring highly on a test of prepotent response inhibition (the “Go/No-Go” task) show far closer correspondence between how much physical activity they intend to take and how much they actually take than those scoring poorly (Hall et al., 2008b). The proportion of variance in physical activity behavior explained by the combination of intentions and EF in the latter study was 59%, almost double the amount typically explained by intentions alone (Sheeran, 2002).

KEY CONCEPT 3 **Temporal Self-Regulation Theory (TST)**

TST proposes three proximal determinants of behavior: intention, prepotency (i.e., “*the likelihood of performance of a given behavior as a function of habit, cues to action, or internal drive states*”), and executive function (Hall and Fong, 2007). These proximal determinants are further influenced by the context in which they are required to be used—different situations will require prepotency and the executive functions to be engaged to varying degrees. Situations where there are short term costs and only long term benefits are hypothesized to require more executive control resources to enact. Additionally, intentions are determined by underlying beliefs and values that have been shaped by personal history and external factors.

In line with TST, research evidence from many different areas and disciplines indicates that EF is predictably related to the enactment and maintenance of a wide range of health behaviors which would be expected to require effortful self-control. Individuals with strong EF (relative to those with weak EF) have been shown to be more likely to stick to their stated dietary intentions (Allan et al., 2011), more likely to enact physical activity intentions (Hall et al., 2008a,b), more likely to regularly attend exercise classes (McAuley et al., 2011), more likely to successfully quit smoking (Brega et al., 2008; Nestor et al., 2011), less likely to drink to excess or develop problems with alcohol (Fernie et al., 2013), less likely to consume fatty foods (Hall, 2012), less likely to give in to dietary temptation (Allan et al., 2010), and more likely to correctly adhere to medication regimes (Stilley et al., 2010, Panos et al., 2014).

Evidence that is particularly indicative of a causal role for EF in determining future health behavior comes from cohort and experimental studies of young adults and adolescents. For example, when young, alcohol-naive adolescents are followed up over a 2-year period, the strength of their executive functioning at baseline significantly predicts when they begin drinking alcohol and whether or not they engage in binge drinking (Peeters et al., 2015). In line with expectations, those with weak EF engage in alcohol consumption sooner than peers with stronger EF suggesting that EF may be required to resist the temptation to engage. Similarly, when adolescents are followed up over a 6-month period, EF prospectively predicts increases in alcohol consumption, but the reverse is not true: alcohol consumption does not prospectively predict decreases in EF (Fernie et al., 2013). This is important, as while it is well-established in older participants that alcohol consumption can lead to cognitive deficits and impairments in EF over time, these results indicate that in adolescence, alcohol consumption, and EF may be associated because weak EF leads prospectively to higher levels of drinking.

Further evidence suggesting a causal relationship between EF strength and health behavior comes from experimental studies using transcranial magnetic stimulation (TMS), a technique where the activity in particular areas of cortex can be reduced using electromagnetic pulses. When TMS is directed at the brain region primarily associated with executive functioning (left dorsolateral prefrontal cortex or DLPFC) to temporarily knock EF “offline,” performance on EF tests decreases and a corresponding reduction in dietary self-control is observed. Specifically, both self-reported snack food cravings and objectively measured snack food consumption are shown to increase in the period immediately following DLPFC stimulation (Lowe et al., 2014). This is in line with fMRI studies of cravings which demonstrate that left DLPFC activation is increased when self-control is exerted (Hare et al., 2009) and is directly associated with the cognitive down-regulation of experienced cravings for both unhealthy foods and cigarettes (Kober et al., 2010).

A wide-range of neuroimaging studies have also linked structural differences in prefrontal regions including cortical thickness, white matter integrity, and gray matter volume to performance on EF tasks (Albinet et al., 2012). These linkages are of particular interest given that age-related structural changes in frontal regions may underpin later life changes in EF which could have implications for health behavior. Indeed, the prefrontal regions that support EF have been shown to be particularly vulnerable to adverse age-related structural changes in a range of gray and white matter properties (Voineskos et al., 2012). Such changes have been proposed to disrupt the connectivity and integrity of functional networks producing a corresponding decline in cognitive functioning (Marstaller et al., 2015). We anticipate that such neurobiologically mediated changes in the efficiency of the EFs may contribute to difficulties in initiating and maintaining healthy patterns of behavior.

## The case for reciprocity

As there is a rapidly accumulating body of evidence supporting both the idea that EF is required to sustain patterns of health-promoting behavior, and that engaging in health behavior improves EF, it is highly likely that the observed relationship is reciprocal. In our own work, we find evidence that when older adults have both their cognitive functioning and physical activity behavior repeatedly assessed over a 6 year period, higher levels of physical activity predict stronger EF from one time point to the next, and stronger EF predicts increases in physical activity form one time point to the next (Daly et al., 2015). Similarly, improvements in EF arising during a physical activity intervention have been shown to predict subsequent exercise adherence 1 year later (Best et al., 2014). It seems that physical activity and EF are synergistic—they improve one another.

As well as being self-reinforcing, the relationship between physical activity and EF may produce additional benefits by fostering improvements in other health enhancing behaviors. This cross-fertilization could occur because the physiological effects of sustained patterns of physical activity lead to improvements in EF which facilitate better planning, initiation, monitoring, and control of other goal-directed health behaviors (Loprinzi et al., 2013, 2015). Whilst we use the example of physical activity, this process could be set in motion by any health behavior that produces changes in the efficiency of the operation of the executive system. Thus even in the absence of intervention there could be cumulative effects whereby health behaviors sustain both EF and each other over time, enhancing healthy functioning, and longevity.

**Figure 2:**
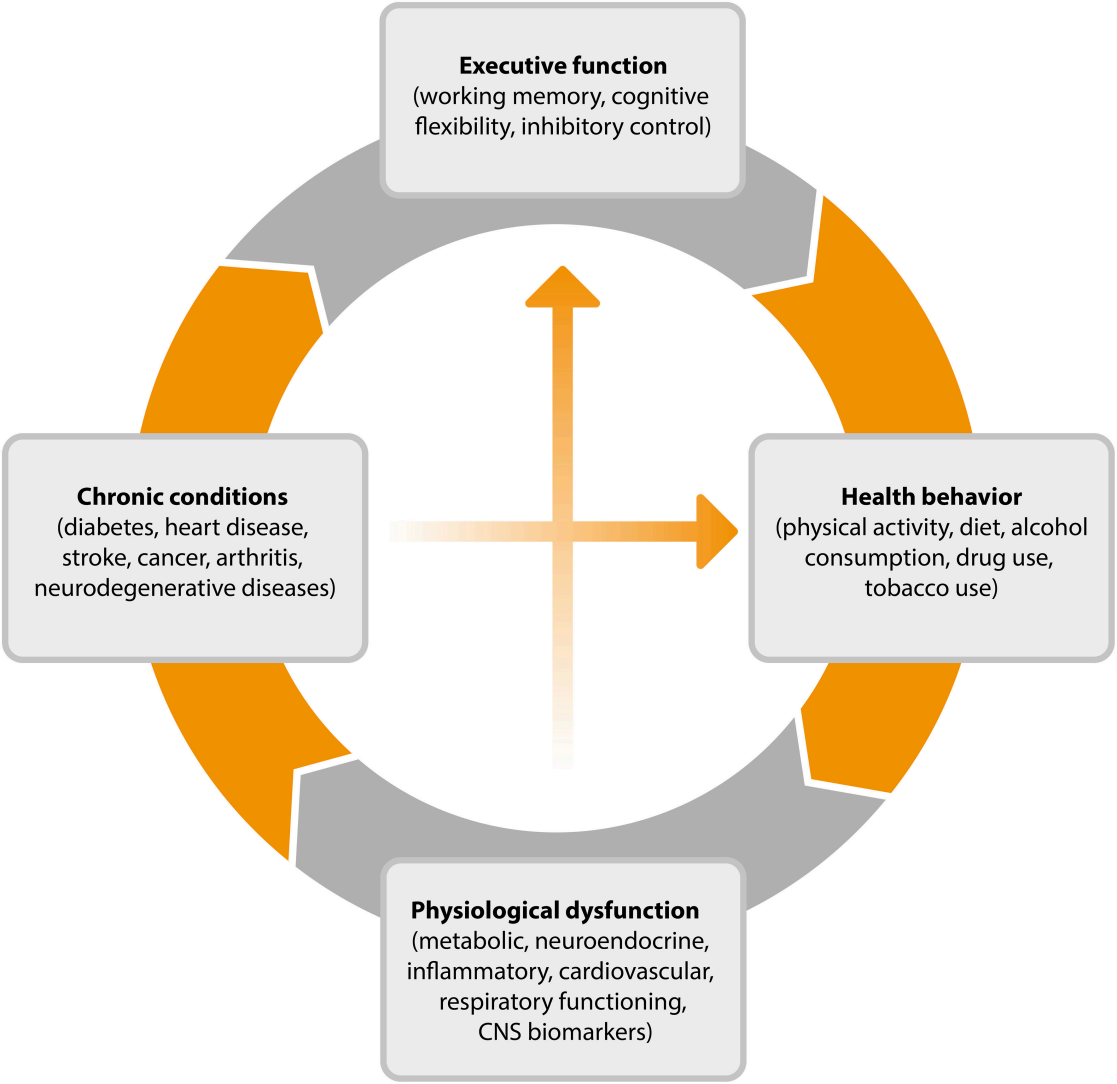
**Cyclical model of the relationship between executive function, health behavior, and disease processes**. CNS, central nervous system.

This cyclical process, outlined in Figure 2, goes some way toward explaining why EF efficiency is related to key health outcomes such as weight gain over time (Guxens et al., 2009), 10-year survival (Duff et al., 2009), and rates of chronic illness (Hall et al., 2010). Individuals with stronger EF will be better equipped, and therefore more likely than others to successfully engage in the types of behavior associated with healthy weight, reduced disease risk and progression, and mortality. By virtue of engaging in these behaviors, their EF will improve, further improving the chance that they will continue to engage in healthy behaviors over time. The existence of such a reciprocal relationship is encouraging from the perspective of intervention development as it suggests that interventions which promote either health promoting behaviors or more efficient EF may have the capacity to produce both cognitive and physical health benefits over time.

## Implications for interventions and the study of aging

If health behaviors and EF are indeed reciprocally linked by a positive feedback loop, the opportunities for, and potential gains of, interventions may be increased markedly. Health behavior change interventions aimed at improving diet, increasing activity levels, reducing smoking, or alcohol consumption would have the potential to produce not only direct benefits for physical and cognitive health, but also to incrementally increase the future likelihood that individuals are capable of maintaining these beneficial behaviors over time.

To give a concrete example: an intervention designed to increase physical activity gives participants a pedometer to wear (e.g., Talbot et al., 2003). Through increased awareness of goal progress and motivation arising from self-monitoring of activity levels, participants manage to increase their daily physical activity levels. A reciprocal relationship between health behavior and EF would suggest that as this new physical activity behavior is enacted, beneficial effects on EF will accrue over time. As EF improves, logically so will the participant's ability to effortfully sustain their new activity behavior, to resist the temptation of inactivity and to deal flexibly with challenges to goal attainment. Positive outcomes in one domain would be expected to beget positive outcomes in the other, thereby progressively strengthening the link between physical activity and EF over time.

Similarly, improvements in EF accrued as a result of cognitive interventions designed to improve EF (e.g., through cognitive training or mindfulness meditation: Ball et al., 2002; Willis et al., 2006) could enable participants to successfully achieve and maintain changes to health relevant behaviors. Regardless of whether an intervention begins with health behavior or cognitive function, the resulting benefits are likely to encompass both domains and to become increasingly apparent over time (assuming the target behavior is maintained). It is already established that interventions designed to improve inhibitory control through cognitive training produce a small but consistently beneficial reduction in health risk behaviors (Allom et al., 2016). Future research needs to assess the ongoing effects of such benefits.

In particular, experimental evidence suggests that a growing set of intervention strategies (e.g., yoga, martial arts, computerized training, meditation) may effectively promote the EFs when they are particularly amenable to change through experience and practice between the ages of 4 and 12 years (Anderson, 2002; Diamond and Lee, 2011; Hsu et al., 2014). Furthermore, quasi-experimental evidence suggests that broader strategies such as increasing the amount of time adolescents spend in formal education may also impact beneficially on cognitive functioning and the EFs specifically (Banks and Mazzonna, 2012; Lager et al., 2016). By producing improvements in the EF during key developmental periods such interventions may help set in motion the proposed cyclical relationships between executive functioning and health behaviors.

It is possible that more efficient EF could directly shape the formation of patterns of health behavior in adolescence leading to long-term consequences for cognitive function. For example, adolescents with a greater inhibitory capacity have been shown to be less likely to start smoking, avoiding what tends to be a lifelong habit with notable adverse consequences for the EFs (Almeida et al., 2011; Mons et al., 2013; Daly et al., in press). It is also possible that EF could indirectly influence health behavior through promoting greater educational attainment which is known to contribute to healthier lifestyles throughout life (Arendt, 2005; St Clair-Thompson and Gathercole, 2006; Bull et al., 2008).

## Conclusions and future directions

The cyclical model of EF and health outlined in Figure 2 shows that health behavior elicits physiological responses that contribute to chronic conditions and ultimately to changes in working memory, cognitive flexibility, and inhibitory control. Numerous studies have documented the protective effect of exercise and of avoiding smoking and excessive alcohol consumption in bolstering cognitive functioning into old age. The potential neurobiological mechanisms underlying these protective effects have also been explicated, particularly in the case of physical activity where animal studies have shed light on the impact of activity on neurotrophin levels, synaptic plasticity, and the growth and development of nervous tissue and blood vessels (see Voss et al., 2013b).

It is only recently that research has begun to uncover the role of EFs in *shaping* complex health behaviors. Such behaviors require planning, sequencing of actions, ongoing monitoring, and adjustment following error detection, and the inhibition of impulsive responses to situational cues, visceral desires and environmental distractions (Mullen and Hall, 2015). When EFs are weak, automatic responses have been shown to predominate and health-enhancing intentions to fail (Allan et al., 2010, 2011). Similarly, individuals with low levels of trait self-control may experience more failures of health-enhancing intentions because they are less likely to actively avoid temptation (Hofmann et al., 2012a). Ultimately, the cumulative effect of lower levels of EF is theorized to be a pervasive inability to execute goal-directed health behaviors resulting in physiological dysfunction and poor health. We propose that this triggers the cycle once again, adversely affecting EF and leading to a self-reinforcing dynamic relationship between EFs and health behaviors over time.

Future research would benefit from further testing of the less well-established pathways in the model outlined in Figure 2. For example, observational studies could be used to test whether a positive feedback loop exists whereby health-enhancing behaviors optimize EF and whether this contributes to the successful maintenance of a healthy lifestyle over time. A range of large scale studies have collected multi-wave data on EF and key health behaviors allowing the suggested mechanistic pathways of the model to be mapped using representative population samples (e.g., Steptoe et al., 2013; Sonnega et al., 2014). These studies often gauge a broad set of biological markers and indicators of disease, allowing the downstream physiological effects of high levels of EF to be charted.

In addition to observational studies, intervention studies that are effective in increasing cognitive control and enhancing facets of EF (e.g., Jaeggi et al., 2008; Diamond and Lee, 2011; Anguera et al., 2013) could be used to identify the influence of EF on health behavior. As such, a virtuous cycle could be identified whereby EF-enhancing interventions may reduce the size of the gap between “good” intentions and health behavior (Allan et al., 2011). By including measures of both health behaviors and intentions to perform those behaviors, EF-enhancing intervention studies could provide a more comprehensive account of the potential positive effects of such interventions. Furthermore, the cyclical model also suggests that intervention studies that are successful in producing sustained improvements in target behaviors such as physical activity or diet (e.g., Astrup et al., 2000; Hunt et al., 2014) could improve engagement in other non-targeted health behaviors by increasing efficient executive control. Incorporating measures of EF and a range of other non-target health behaviors into interventions targeting a specific health behavior would allow this possibility to be tested.

Given the current situation where the age-related decline in EF impairs the ability of older people to live independently, evidence generated from such studies would help establish the nature of the reciprocal relationship between EF and health behavior and lay the groundwork for an integrated approach to the development of empowering interventions that simultaneously promote cognitive enhancement and healthy aging.

## Author contributions

JA, DM, and MD all made substantial contributions to the intellectual content of the work and were actively involved in drafting and revising the manuscript. All authors approved the final version of the manuscript and agree to be accountable for all aspects of the work.

### Conflict of interest statement

The authors declare that the research was conducted in the absence of any commercial or financial relationships that could be construed as a potential conflict of interest.
